# On the possibility of using X-ray Compton scattering to study magnetoelectrical properties of crystals

**DOI:** 10.1107/S2053273316000863

**Published:** 2016-02-16

**Authors:** S. P. Collins, D. Laundy, T. Connolley, G. van der Laan, F. Fabrizi, O. Janssen, M. J. Cooper, H. Ebert, S. Mankovsky

**Affiliations:** a Diamond Light Source Ltd, Harwell Science and Innovation Campus, Didcot, OX11 0DE, England; b Department of Physics, New York University, New York, NY 10003, USA; c Department of Physics, University of Warwick, CV4 7AL, England; d Universität München, Department Chemie, Haus E2.033, Butenandtstrasse 5-13, D-81377 München, Germany

**Keywords:** Compton scattering, magnetoelectric properties, synchrotron radiation, density functional theory, DFT, Korringa–Kohn–Rostoker (KKR) Green’s function method, KKR method

## Abstract

This paper discusses the possibility of using Compton scattering – an inelastic X-ray scattering process that yields a projection of the electron momentum density – to probe magnetoelectrical properties. It is shown that an antisymmetric component of the momentum density is a unique fingerprint of such time- and parity-odd physics. It is argued that polar ferromagnets are ideal candidates to demonstrate this phenomenon and the first experimental results are shown, on a single-domain crystal of GaFeO_3_. The measured antisymmetric Compton profile is very small (≃ 10^−5^ of the symmetric part) and of the same order of magnitude as the statistical errors. Relativistic first-principles simulations of the antisymmetric Compton profile are presented and it is shown that, while the effect is indeed predicted by theory, and scales with the size of the valence spin–orbit interaction, its magnitude is significantly overestimated. The paper outlines some important constraints on the properties of the antisymmetric Compton profile arising from the underlying crystallographic symmetry of the sample.

## Abstract 2

The possibility of using X-ray Compton scattering to reveal antisymmetric components of the electron momentum density, as a fingerprint of magnetoelectric sample properties, is investigated experimentally and theoretically by studying the polar ferromagnet GaFeO_3_.

## 1. Background   

Compton scattering provides a projection of the electron momentum distribution in a target material (Cooper, 1985 ▸). While the exact relativistic form of the differential scattering cross section is complex, the momentum density derived from every measurement, and calculated by every theory, to date, has been symmetric. We argue that this is because all materials investigated so far have been symmetric with respect to time reversal or spatial inversion. Materials whose orbitals possess neither symmetry are said to be magnetoelectric as they play a major role in magnetoelectric phenomena. Of particular interest are toroidal moments, corresponding to time- and parity-odd vectors, that not only play a vital role in magneto­electric phenomena (Spaldin *et al.*, 2008 ▸) but have been suggested to be implicated in high-*T*_c_ superconductivity (Scagnoli *et al.*, 2011 ▸).

It is therefore of considerable interest to identify novel experimental probes of such moments. We show that the antisymmetric Compton profile is a unique signature of magnetoelectric properties and should therefore provide a very sensitive probe of the underlying orbitals, that can be compared in detail to electronic structure calculations to elucidate the underlying physics. In this article, we outline the principles behind this phenomenon, examine the possibility of observing such an effect in the polar ferromagnetic crystal GaFeO_3_, describe an experiment to measure the antisymmetric Compton profile, and compare the results with relativistic first-principles calculations.

## 2. Compton scattering and the electron momentum density   

X-ray Compton scattering is an inelastic scattering process whereby the energy loss is an almost linear function of a projection of the electron momentum density: 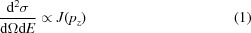
where 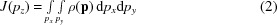
is called the Compton profile (Cooper, 1985 ▸). Here, 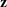 lies (almost) parallel to the momentum transfer 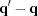 and 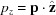 is the *z* projection of electron momentum. The momentum density 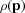 and Compton profile 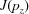 are historically considered to be symmetric with respect to reversal of the momentum variable 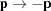 (or 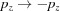). We suggest that this need not be the case.

Let us first discuss the conditions under which the momentum density is symmetric. Since momentum is a function of both space and time (classically, 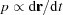) we find that *either* inverting 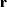 (parity inversion) *or* *t* (time reversal) inverts 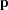 (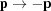). Consequently, any parity-even (centrosymmetric) or time-even (non-magnetic) material must satisfy 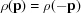. Compton profiles of this dominant class of materials are always symmetric.

However, no such constraint applies to materials that lack both time and inversion symmetry. Moreover, such systems form an interesting and important class of materials that often exhibit magnetoelectric phenomena such as linear magnetoelectric coupling, destined to play a key role in future technologies (Spaldin & Fiebig, 2005 ▸). We are therefore alerted to the possibility of using Compton scattering as a probe of time- and parity-odd magnetoelectric phenomena.

It is worth noting that while the breakdown of the impulse approximation (IA) can lead to an asymmetry in the measured Compton profile (Huotari *et al.*, 2001 ▸), such effects need not be considered for the current analysis. This is partly because our measurements are not of the asymmetry in the energy spectrum directly, but rather of the intensity difference that is caused by an asymmetry in the electron momentum distribution. Moreover, the orbitals that are expected to contribute to the effect discussed here are relatively low-energy valence states, whereas the breakdown of the IA is expected to affect mainly tightly bound core electrons.

It is convenient to write the total momentum density as the sum of a symmetric component (with respect to 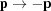) and an antisymmetric part: 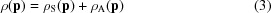
giving 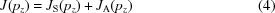
where 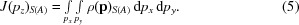
The quantities 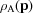 and 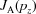 represent time- and parity-odd properties. Since Compton scattering is an incoherent process, these objects are averages over all the constituent orbitals and therefore governed by the crystal (magnetic) point-group symmetry.

## 3. Zero-sum rule   

If there is no net flow of electrons in the sample the integral of the flow along positive and negative *z* directions must cancel, *i.e.*

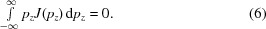
While this is satisfied trivially for the symmetric Compton profile, it imposes a useful constraint on each half of the antisymmetric profile: 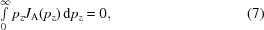
thus providing a ‘zero-sum rule’ that can be used to verify experimental results and model calculations. Most trivially, the zero-sum rule dictates that, for each half of the asymmetric profile, the existence of a positive contribution implies the existence of a negative one, and *vice versa*.

## 4. Feasibility argument: classical orbitals   

**Figure 1:**
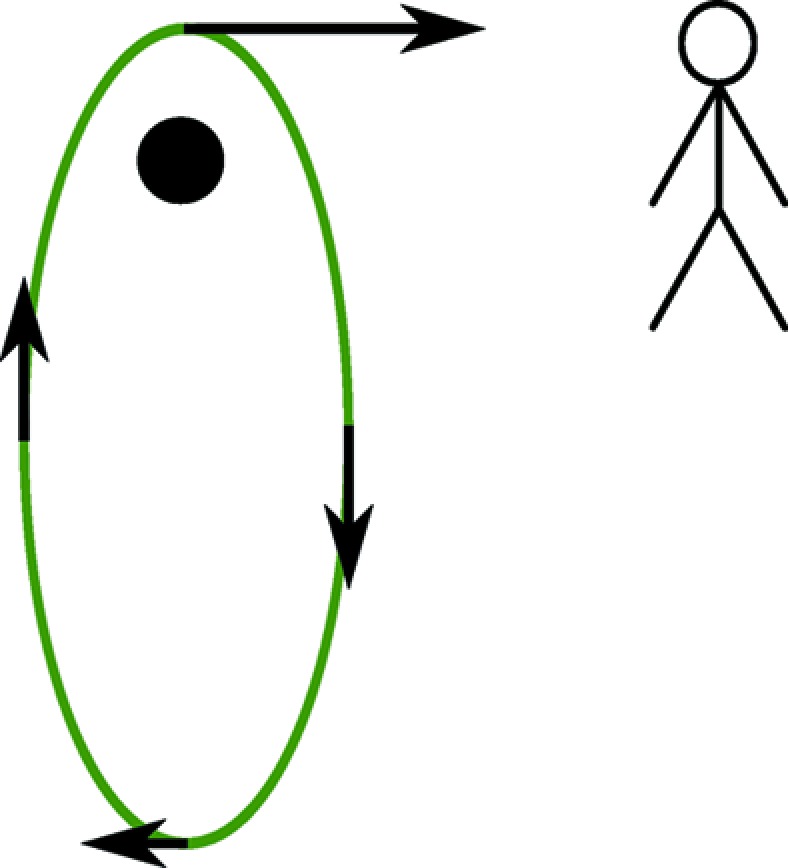
A *gedankenexperiment* to demonstrate the possibility of a non-symmetric momentum density in a classical orbital. An observer sits in the plane of a highly eccentric planetary orbit, perpendicular to its major axis, and with the planet moving towards the observer at perihelion. The observer measures the amount of time each projection of the planet’s momentum is observed for (perhaps *via* a Doppler-shift measurement) during a complete orbit, observing a large positive momentum projection for a small amount of time, and a small negative momentum for a long time as the planet orbits furthest from the star. The largest positive momentum has no negative counterpart and so the momentum density is clearly asymmetric. It is clear that this function is reversed under reversal of time (*i.e.* the planet orbits in reverse), and also under spatial inversion, realized (in this two-dimensional system) by a rotation of π of the orbital within the orbital plane.

Finding no obvious symmetry arguments for momentum densities and Compton profiles to be symmetric is, of course, far from demonstrating that they are not. However, one can employ a simple thought experiment to see that such an asymmetry is present in classical orbitals. Consider a highly elliptical planetary orbital, observed from within the orbital plane, as shown in Fig. 1 ▸. The orbiting body would be seen to have a very large positive momentum projection (towards the observer) for a short period of time, when the orbiting ‘planet’ is closest to the ‘star’ that it orbits. Conversely, it would exhibit a small negative momentum projection for a long period of time when it is far from the star and moving slowly. The large positive momentum has no negative counterpart and so the momentum projection distribution (analogous to the Compton profile) *must* be asymmetric. Note also that such an orbital is asymmetric with respect to time reversal and spatial inversion: reversing time would reverse the direction of the orbit, and spatial inversion would reverse its eccentricity.

## 5. Momentum density and rotational properties   

The electron momentum density is a real-valued function of momentum, 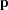, or (equivalently) of its magnitude, *p*, and direction, 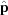. We can expand this density in terms of a complete set of angular functions and prefactors that depend on *p*. For example, 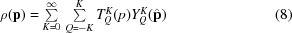
where 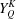 are real spherical harmonics (also referred to as multipoles or spherical tensors) of rank *K* and projection *Q* (Lovesey *et al.*, 2005 ▸), and 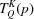 are the corresponding tensor components and are functions of *p*. The merit of such an expansion lies in the fact that each non-vanishing multipole 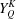 must be consistent with the symmetry of the physical system. For example, an isotropic system allows only a single term in the expansion and we find 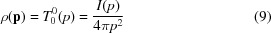
where 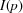 is the radial momentum distribution (Cooper, 1985 ▸).

For the present study we are primarily interested in the antisymmetric momentum density: 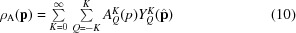
where 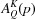 are the corresponding tensor components. Since reversal of the momentum vector, 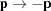, is equivalent to carrying out the rotations 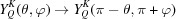, and all *K* = odd (even) real spherical harmonics are odd (even) under this transformation, we conclude that the antisymmetric momentum density contains only *K* = odd terms, ruling out contributions from magnetic monopoles or quadrupoles. (Conversely, the symmetric density contains only even multipoles, including the scalar *K* = 0 term). We can therefore write 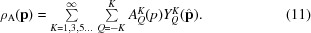
Importantly, the lowest-order allowed component has *K* = 1 and is therefore associated with a parity-odd, time-odd vector, *i.e.* a toroidal moment (Lovesey *et al.*, 2005 ▸). A non-vanishing antisymmetric Compton profile therefore requires a material whose point-group symmetry permits the existence of odd-rank time- and parity-odd multipoles. One might expect systems that allow the lowest (*K* = 1) rank multipole to be most favourable. If we assume that the antisymmetric momentum density is dominated by this term then we have 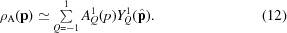
Furthermore, if the direction of the toroidal moment is fixed by symmetry (*i.e.* the same for all momentum magnitudes) then the radial and angular parts can be factorized: 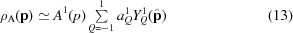
(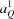 are now constants), which can be written in Cartesian form as 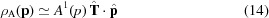
where 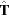 is the toroidal moment direction.

The Compton profile of such a momentum density can be written: 
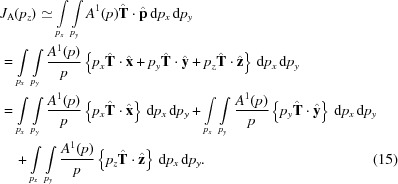
The first two terms vanish as they involve integrals over functions that are odd with respect to the integration variable. This leaves 
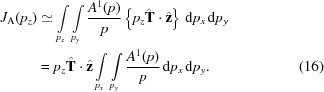
While the integral is not straightforward to compute, there are two noteworthy aspects of this result. First, the Compton profile changes sign with 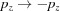, as expected. More interestingly, the Compton profile scales with the *z* projection of the toroidal moment unit vector. The latter gives a complete description of the angular dependence of the antisymmetric Compton profile in the case where multipoles of 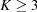 are negligible: the signal is maximum when the toroidal moment is along *z*, of equal and opposite magnitude when antiparallel, and zero when perpendicular. Armed with this approximate form, and the exact ‘zero-sum rule’ in equation (7), a clear picture begins to emerge about what the antisymmetric Compton profile should look like.

## 6. Experimental verification – suitable materials   

It is likely that the antisymmetric part of the Compton profile is small as it depends on a subtle aspect of the anisotropy of the orbital polarization. An ideal experiment to test these ideas is therefore one where the antisymmetric part can be reversed simply and rapidly, inducing the smallest possible systematic error and allowing a sensitive ‘difference’ measurement to be performed. Reversal of the toroidal vector in many magnetoelectric materials requires simultaneous application of electric and magnetic fields, typically applied during ‘field cooling’. An interesting class of materials where the toroidal moments are more easily manipulated are polar ferromagnets. One such material, that has been studied with X-rays for its directional dichroism (Kubota *et al.*, 2004 ▸) and magnetoelectric multipoles (Staub *et al.*, 2010 ▸), is GaFeO_3_. Large polar single crystals are available and the ferromagnetic moment can be reversed with a modest applied field. GaFeO_3_ orders magnetically at the relatively high temperature of *T*_C_ ≃ 210 K. We therefore selected GaFeO_3_ as a potentially suitable test material and consider next the implications of crystal symmetry on the observable physical phenomena.

## 7. GaFeO_3_: symmetry and allowed tensor components   

GaFeO_3_ (space-group No. 33, 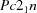) is a polar ferromagnet. It possesses both a magnetic (axial, time-odd) and polar (time-even) vector moment. We have discussed the need for time- and parity-odd multipoles in the context of antisymmetric Compton profiles, and the desirability to possess a toroidal (polar, time-odd) moment. We now apply the magnetic crystal symmetry to all four permutations of time/parity odd/even vectors in order to find out (i) if they can exist and (ii) in which direction(s) they may point. The crystal point-group symmetry in the high-temperature paramagnetic phase is *mm*2. We can denote the symmetry group as 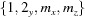 (the identity, twofold rotation about *y*, mirrors normal to *x* and *z*). There are several possible magnetic groups that are consistent with this point group, which are formed by taking each spatial symmetry operator and either applying time reversal or not. Four such groups can be generated, with each placing specific constraints on the directions of the possible vectors, or rendering them absent.

For GaFeO_3_, it is known that the magnetic easy axis lies along *x* (crystal *a* axis). However, the anisotropy is not strong and it is informative to consider the properties of all possible magnetic symmetry groups. These are shown in Table 1 ▸. The procedure for analysing the properties of various tensors, permitted by crystal symmetry, is described in Collins & Bombardi (2010 ▸). Briefly, the process involves generating a random vector, odd or even under *T* (time) and *P* (parity), generating a transformed vector for each of the four symmetry operations in the magnetic group, and adding the four resulting vectors. We find that the resultant, due to the high symmetry of the system, is always either zero or lies parallel to one of the three Cartesian/crystal axes.

**Figure 2:**
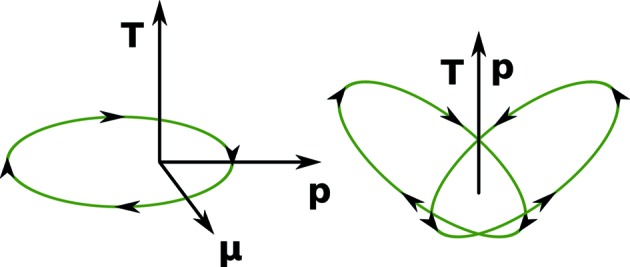
Left: a classical representation of the dipole (vector) moments permitted by a classical orbital. The magnetic moment 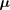 points out of the page and is perpendicular to the orbital plane. The polar (electric dipole) moment, 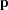, lies parallel to the major orbital axis. The toroidal moment, **T**, lies perpendicular to the polar and magnetic moments, in the direction of their cross-product, and is thus odd under reversal of either, but even under reversal of both. Right: the slightly counter-intuitive case of zero magnetic moment and parallel polar and toroidal moments can be visualized classically as the superposition of two rotated and reflected orbitals, forming a ‘butterfly’ pattern.

Several interesting points emerge from this symmetrization procedure. First, we see that in all cases the time-even polar vector lies along *y*. This makes sense as only the magnetic symmetry is changed between the four groups. We see that there is no time-even axial vector. The four magnetic groups support a magnetic vector along 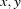 and *z* for the first three, with the fourth group not supporting a net magnetic moment. Of particular interest are the toroidal vectors. For the first and third groups (the homomorphic 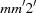 and 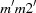 groups), the toroidal vector is perpendicular to the magnetic and polar vector, consistent with the sketch in Fig. 1 ▸. The second group (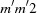) does not allow a toroidal vector. Interestingly, a toroidal vector is allowed by the fourth group (*mm*2), despite the absence of a magnetic moment, and it is *parallel* to the polar vector. This slightly counter-intuitive scenario can be visualized as the sum of two such classical orbitals, resembling a butterfly (Fig. 2 ▸).

**Figure 3:**
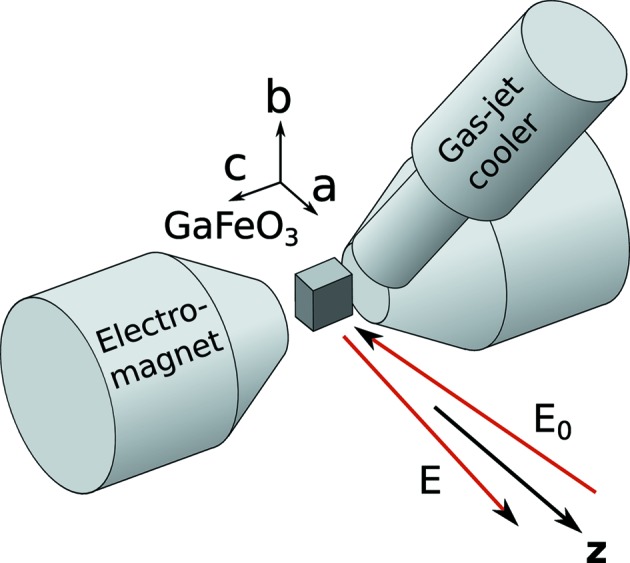
A schematic depiction of the experimental setup designed for measuring the antisymmetric Compton profile in a crystal of the polar ferromagnet GaFeO_3_. A reversible magnetic field was applied along the crystal *c* axis, with the polar *b* axis vertical. The toroidal *a* axis was aligned close to the direction of photon momentum transfer, which defines the projection direction for the momentum density. The sample was held below its magnetic ordering temperature by a nitrogen gas-jet cooler, and scattered photons detected by a multi-element germanium solid-state detector array (not shown), close to back-scattering.

All four symmetry groups support 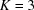 and higher (odd) rank multipoles. For the present case, where the magnetic field is applied along the magnetic easy axis (the first magnetic group in the table), the presence of a toroidal moment suggests that GaFeO_3_ is a suitable material for observing an antisymmetric Compton profile, and that the *c*-axis toroidal moment should be directed along the momentum transfer (*z* axis), with the polar *b* axis and magnetic *a* axis both perpendicular, as shown in Fig. 3 ▸.

## 8. Experiment on GaFeO_3_   

Experiments were carried out on beamline I12 (Diamond Light Source), using a linearly polarized monochromatic incident X-ray beam of energy 125 keV and bandwidth of 0.6 keV, selected by controlled bending of a double Laue monochromator (Drakopoulos *et al.*, 2015 ▸). Compton scattering was detected close to back-scattering (2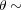 169°) by a 23-element germanium solid-state detector. The orientation of crystal (a single polar domain – see Appendix *A* [App appa]), X-ray beams and magnetic field were as shown in Fig. 3 ▸ and the sample was maintained at a temperature of 100 K with a nitrogen gas-jet cooler. As the aim of the experiment was to observe a small (antisymmetric) difference in the Compton profiles measured with two opposite magnetic field directions, the (0.3 T) field was flipped rapidly and repeatedly (1 s counting time for each direction) while data were accumulated for around 48 h.

**Figure 4:**
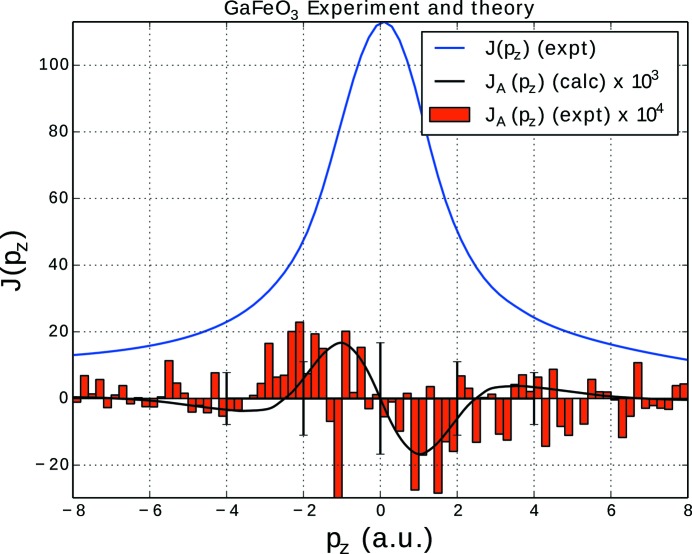
Experimental Compton scattering results from GaFeO_3_. The total electron momentum distribution (Compton profile) is shown in blue, normalized such that the integral is the total number of electrons in the unit cell (eight GaFeO_3_ formula units). The red bars show the antisymmetric Compton profile derived from the difference in Compton profiles measured with opposite magnetic field directions. Error bars are shown in black. Also shown on the plot is the calculated antisymmetric profile, convoluted with a Gaussian of width 0.8 a.u., to mimic the experimental momentum resolution. Note that, although the calculated and measured line shapes look similar, the experimental differences are of the same order as the statistical errors (error bars), and that the measurements are scaled by an order of magnitude compared to the calculations.

The experimental Compton scattering results are shown in Fig. 4 ▸. The total Compton scattering (electron momentum density) is shown in blue, with the magnetic ‘difference’ signal indicated by red bars. The difference data have been multiplied by 10^4^, indicating that the difference, and any competing systematic and random errors, are extremely small. While the difference profile gives a hint of the anticipated antisymmetric shape, the effect is of the same order as the statistical errors (black bars). As such, the experimental results do not show conclusive evidence of the predicted asymmetry but give a clear indication of the maximum magnitude of such an effect.

## 9. First-principles calculations on GaFeO_3_   

To confirm the occurrence of an antisymmetric Compton profile in polar ferromagnets, density functional theory (DFT)-based theoretical investigations have been performed. The Compton profile was calculated from first principles using the Korringa–Kohn–Rostoker (KKR) Green’s function method. This implies the electronic Green’s function 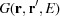 is represented by means of the multiple scattering formalism by 
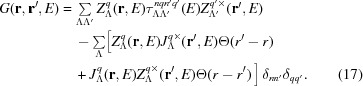
Here 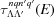 is the scattering path operator with the combined index 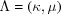 representing the spin–orbit and magnetic quantum numbers κ and μ, respectively (Rose, 1961 ▸), and 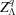 and 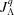 are the four-component regular and irregular solutions, respectively, to the single-site Dirac equation for the atomic site *q* (Ebert *et al.*, 2011 ▸). The superscript 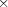 indicates the left-hand-side solution of the Dirac equation. The electron momentum density ρ(**p**) = 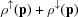 is decomposed into its spin-projected components 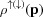, which are given by the Green’s function represented in momentum space, 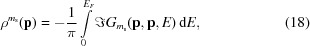
where 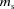 represents the spin character. 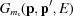 is expressed in terms of the real-space Green’s function 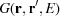 as follows: 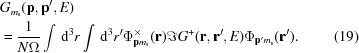
Here Ω is the volume of the unit cell and 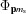 are the eigenfunctions of the momentum operator, which can be written as 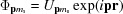, where 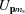 is a four-component spinor satisfying the equation (Rose, 1961 ▸) 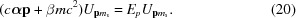
Using a Rayleigh-like expression, one obtains the angular momentum expansion for the eigenfunctions (Benea *et al.*, 2006 ▸), 
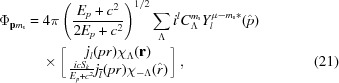
where 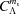 are Clebsch–Gordan coefficients, 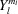 are complex spherical harmonics, 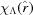 are spin-angular functions and 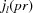 are spherical Bessel functions.

The electronic structure calculations have been performed using the fully relativistic multiple scattering KKR Green’s function method (Ebert *et al.*, 2011 ▸, 2012 ▸) (adopting the atomic sphere approximation, ASA). Exchange and correlation were treated within the framework of local spin density approximation (LSDA) using the parametrization of Vosko, Wilk and Nusair (Vosko *et al.*, 1980 ▸). Chemical disorder due to intermixing between the Fe and Ga sublattices in the system is treated by means of the coherent potential approximation (CPA) alloy theory (Soven, 1967 ▸; Ebert *et al.*, 2011 ▸). For the angular momentum expansion of the Green’s function [see equation (17)] a cutoff of 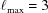 was applied. 

**Figure 5:**
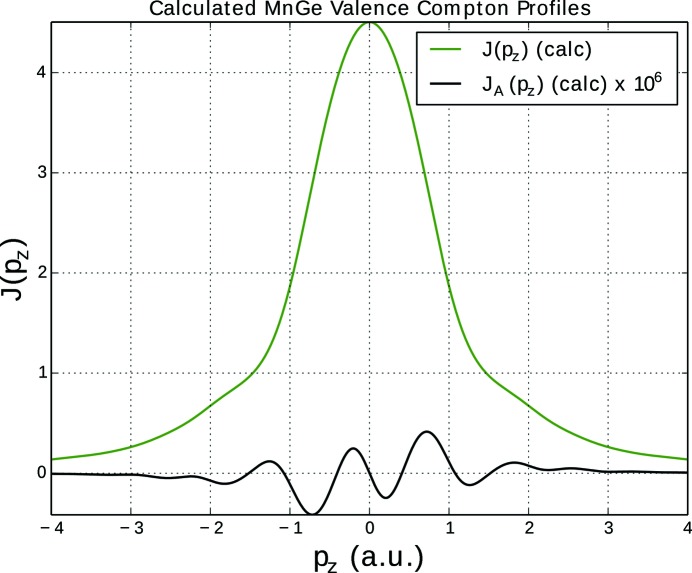
Calculated total valence electron (green) and antisymmetric (black) Compton profiles of the chiral magnet (parity- and time-odd) MnGe, broadened by 0.2 a.u. Calculations are done for the ferromagnetically ordered system with a magnetic field direction parallel to [1, −1, 0] and momentum transfer vector 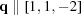.

As a first step in the investigations of the occurrence of the antisymmetric Compton profile, the calculations have been performed for the non-centrosymmetric compounds MnGe and FeGe, with B20 structure (space group 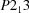). FeGe is ferromagnetically ordered at ambient pressure with a Curie temperature 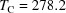
 K (Wilhelm *et al.*, 2012 ▸), while MnGe can be synthesized under a high pressure and exhibits antiferromagnetic (AFM) order below 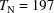
 K with a saturated magnetic moment of 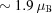/Mn at 5 K (Kanazawa *et al.*, 2011 ▸). Despite that, the calculations for both compounds have been performed for an FM (ferromagnetic) alignment of the magnetic moments to fulfil the precondition for the observation of an antisymmetric Compton profile. The calculated Mn magnetic moment in MnGe, of 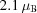, fits rather well the experimental results. In line with the experimental setup, the orientation of the magnetization was taken to be perpendicular to the sample threefold axis as well as to the momentum transfer vector 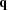. For both systems the antisymmetric part of the calculated Compton profile is very weak (but still significant), as is demonstrated in Fig. 5 ▸ showing the results for MnGe.

These results are in line with the results of the measurements on MnSi with B20 structure, performed with the same geometry and demonstrating the magnitude of the antisymmetric Compton profile that is beyond the current accuracy of the experiment.

In the case of the GaFeO_3_ system the calculations were performed taking the occupation numbers 0.18 and 0.35 for Fe atoms on Ga1 and Ga2 sites [Fe(Ga1) and Fe(Ga2)] and 0.77 and 0.7 for the Fe1 and Fe2 (Fe sites), respectively. The structure and composition information was taken from the available experimental data. The Fe magnetic moments obtained in the calculations are *M*_Fe(Ga1)_ = 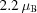, *M*_Fe(Ga2)_ = 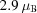, *M*_Fe1_ = 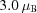 and *M*_Fe2_ = 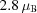, with the Fe(Ga1) and Fe1 magnetic moments antiferromagnetically oriented to Fe(Ga2) and Fe2, respectively.

**Figure 6:**
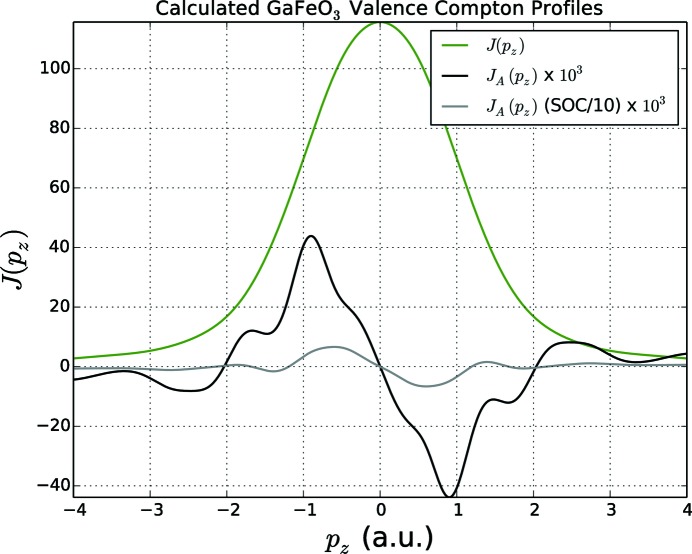
Calculated GaFeO_3_ total valance electron (green) and antisymmetric (black) Compton profiles, broadened by 0.2 a.u. (a quarter of the momentum resolution of the present work), indicating the potential benefit of performing measurements with improved resolution. Also shown is the same antisymmetric profile, but calculated with the spin–orbit coupling (SOC) reduced by a factor of ten. The antisymmetric profile is reduced by very nearly the same factor, showing that SOC plays an essential role in the underlying physics.

The calculations of the antisymmetric Compton profile have been performed for the geometry as used in the experiment. This implies that the photon momentum transfer is along the toroidal axis *a*, while the magnetic field lies parallel to the crystal *c* axis. Assuming that the total magnetic moment follows the direction of the magnetic field, the contribution of the valence electrons to the Compton profile has been calculated for two opposite orientations 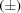 of the magnetization. Accordingly, the antisymmetric Compton profile 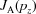 is defined as the difference 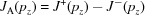. Restricting the calculations to the valence electrons implies that contributions to 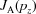 by core electrons are negligible. This simplification is very well justified. The total Compton profile due to the valence states is shown in Fig. 6 ▸ together with the antisymmetric Compton profile, calculated using a momentum broadening of 0.2 and 0.8 a.u. The latter value corresponds to the experimental momentum broadening. As can be seen, the amplitude of the antisymmetric profile is about three orders of magnitude smaller than for the total Compton profile. In the experiment this difference is even more pronounced, as can be seen in Fig. 4 ▸. To account for the rather low experimental momentum resolution of about 0.8 a.u., a corresponding momentum broadening has been applied to the calculated Compton profiles, shown in Fig. 4 ▸ (black line). This results in particular in a substantial decrease of the amplitude bringing the theoretical results closer to the experiment. Another source for the apparent overestimation of the antisymmetric part of the Compton profile in the calculations is the finite temperature of the measurements (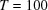
 K). Taking into account the rather small critical temperature 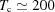
 K, one can expect a rather pronounced temperature-induced magnetic disorder in the system which should lead to a smearing of the electronic states and as a result to a decrease of the magnitude of the antisymmetric Compton profiles.

As the antisymmetric Compton profile is a consequence of the anisotropy of the orbital polarization and accordingly is first of all a relativistic effect, it should depend on the strength of spin–orbit coupling (SOC) in the system. To demonstrate this, the calculations have been performed with the SOC scaled. In Fig. 6 ▸ the antisymmetric profile obtained using a scaling factor 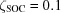 is plotted together with that obtained without any SOC scaling. One can clearly see a decrease of the amplitude of the profile by nearly one order of magnitude due to the SOC scaling. A further decrease of the scaling factor leads to a collapse of the antisymmetric part of the Compton profile.

**Figure 7:**
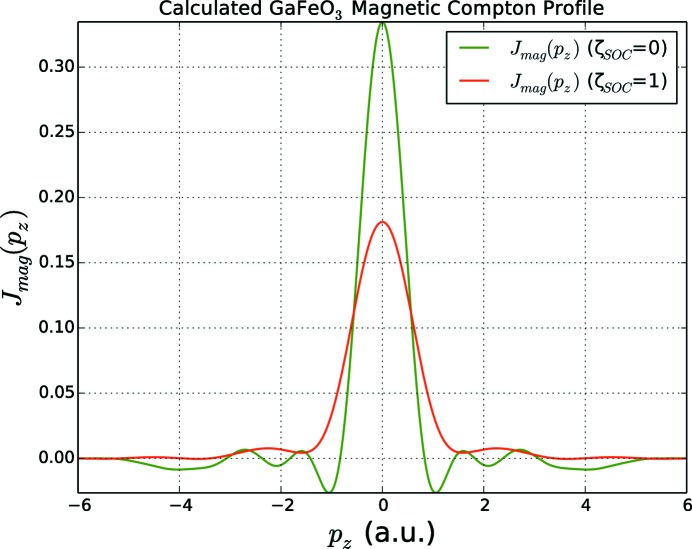
Magnetic Compton profile for GaFeO_3_: red line with full SOC and green line with SOC suppressed. A momentum broadening of 0.2 a.u. has been applied. Both profiles have been normalized to the total magnetic moment of 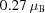 per formula unit.

Note also that spin–orbit interaction has a rather pronounced effect on the shape of the *magnetic* Compton profile (MCP) – the spin-projected momentum density. Fig. 7 ▸ gives the MCP for GaFeO_3_ calculated without (green line) and with (red line) SOC. As one can see, neglect of the SOC results in an increase of the amplitude at 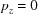
 a.u., as well as a more pronounced oscillatory momentum dependence.

## 10. Conclusions and future prospects   

We propose a new class of Compton scattering experiment with the potential to provide the antisymmetric part of the electron momentum density in materials. We show that the antisymmetric Compton profile is a unique fingerprint of time- and parity-odd properties of the underlying orbitals, and thus a sensitive probe of magnetoelectric phenomena. Initial experiments on the polar ferromagnet GaFeO_3_ demonstrate that our experimental technique is extremely sensitive, leading to very small systematic errors. Our results show that the magnitude of the antisymmetric momentum density, after broadening with the experimental momentum resolution of 0.79 a.u., is not larger than around 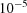 of the peak in the symmetric part. While the optimistic eye might be tempted to pick out an antisymmetric difference signal (Fig. 4 ▸) above the statistical noise, we cannot claim that the present results are conclusive in this respect.

The main scientific motivation for these experiments is to provide a sensitive and stringent test of first-principles electronic structure calculations. To this end, we have performed calculations of the antisymmetric Compton profile using the KKR Green’s function method. The results of these calculations suggest that the antisymmetric profile should be far larger than was observed in the measurements, thus proving that the antisymmetric Compton profile is indeed a highly challenging and stringent test of competing theories. Moreover, even without comparison to experimental data, deficiencies in the theory are evident from the fact that the calculated profiles clearly (visually) violate our zero-sum rule.

Deficiencies in the experimental determination of this effect are even more dramatic than those of the theory. The X-ray detection efficiency, determined by the total detector solid angle, is 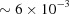. Moreover, the difference signal is inevitably washed out by convolution with the momentum resolution, determined by the incident photon beam bandwidth, and detector energy and angular resolution. Technological developments, especially high-resolution high-energy photon detector arrays, are likely to improve the quality of experimental data by a very significant factor, rendering such studies straightforward in the future.

To conclude, we have shown that antisymmetric Compton scattering should exist in an interesting and topical class of material. First attempts to measure this have shown that it is extremely small and close to the limit of statistical uncertainty, but differs sufficiently from predictions of state-of-the-art first-principles calculations, to provide a very sensitive test of the microscopic origins of magnetoelectric phenomena. We expect that future improvements in experimental technology will make such measurements more straightforward. The present study focuses on a polar ferromagnet. While such a system affords simple control over magnetoelectric polarization, *via* magnetic field flipping, we note that this is by no means necessary. Antisymmetric components of the electron momentum density should be observable in materials that are odd under time and parity reversal separately, but even under the combination of the two, such as an antiferromagnetic/antiferroelectric crystal.

Finally, it is perhaps worth noting that Compton scattering has the potential to probe other exotic polarization-dependent properties. For example, one could envisage a study of surface states in a topological insulator, using the spin sensitivity of magnetic Compton scattering (Cooper, 1985 ▸) to probe the correlation between the momentum vector of the wavefunction and its spin direction. Such experiments would be significantly more challenging than the present one, due to the required surface sensitivity and the reduced cross section for magnetic Compton scattering, but might be feasible in the future.

## Figures and Tables

**Figure 8:**
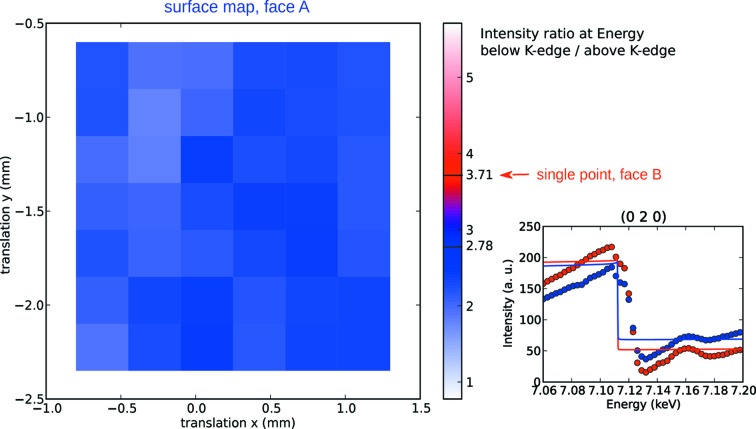
Left: a map of the intensity ratio between photon energies 7.10 and 7.16 keV (below and above the Fe *K* edge), on the (0 2 0) reflection, for one sample face. The red arrow indicates the intensity ratio for the opposite face, in the central position of the map. The values 2.78 and 3.71, highlighted in the colour bar, represent the expected ratios of a monodomain state, for the two faces, respectively. Right: the complete energy profiles of the reflection, measured at the centre of the map on the two opposite faces (red and blue). The dots represent experimental data (integrated rocking curves); the lines are simulated from a simple model of resonant diffraction from isolated Fe^3+^ ions.

**Table 1:** Allowed vectors and their directions, for various magnetic symmetry groups (+/− indicates the absence/presence of the time-reversal operator) and vector symmetry with respect to time (*T*) and parity (*P*) The Cartesian axes 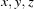 are parallel to the crystal axes 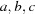. The third of the four listed groups, 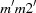 (conventionally written 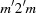), corresponds to the symmetry of GaFeO_3_.

Symmetry name	Symmetry operation	*T*	*P*	Vector type	Vector direction
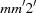	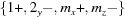	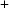	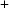	Axial (time-even)	0
	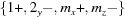	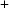	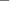	Polar	*y*
	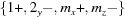	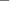	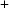	Magnetic	*x*
	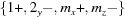	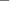	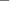	Toroidal	*z*
					
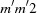	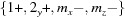	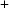	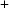	Axial (time-even)	0
	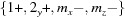	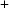	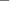	Polar	*y*
	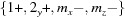	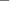	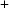	Magnetic	*y*
	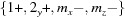	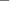	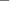	Toroidal	0
					
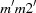	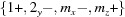	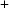	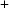	Axial (time-even)	0
	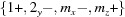	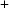	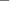	Polar	*y*
	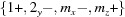	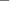	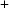	Magnetic	*z*
	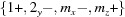	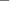	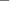	Toroidal	*x*
					
*mm2*	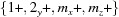	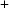	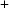	Axial (time-even)	0
	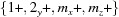	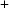	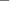	Polar	*y*
	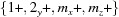	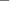	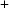	Magnetic	0
	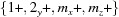	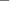	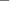	Toroidal	*y*

## Appendix A Verification of the single polar domain state in the GaFeO_3_ crystal

Since the antisymmetric Compton profile would be expected to vanish if the X-ray beam sampled an equal population of opposite polar domains, the experiment hinged on being confident that the entire crystal, of dimensions ∼3 × 3 × 3 mm, consisted of a single polar domain.

As the crystal was far too large to establish its polar properties by conventional X-ray diffraction techniques, we employed a novel approach, described by Fabrizi *et al.* (2015 ▸), whereby spatial diffraction maps are made of opposite sample faces at two photon energies, just above and below the Fe *K* edge.

First, the detailed energy dependence of the (0 2 0) reflection was collected between 7.06 and 7.2 keV, in the central position of the surface, for the two opposite faces. It is expected, given the geometry of the system, that reversing the face of the sample equates to reversing the sign of the polar vector, if the crystal is in a monodomain state. This is confirmed by the difference in the two energy profiles (Fig. 8 ▸). The contrast between polar states is provided by the resonant contribution of the X-ray diffraction, which is enhanced in the proximity of an atomic absorption edge.

To verify that the monodomain state extends to the whole sample, a spatial map was collected on one of the faces, by measuring the intensity ratio between two opportune photon energies (7.10 and 7.16 keV). This provides us with a fingerprint of the domain composition, irrespective of the overall scattering power of the specific portion of the crystal illuminated by the X-rays.

These measurements confirmed that each face exhibited a single polar domain, and that the polar vector reversed between opposite faces. It therefore seems extremely likely that the entire crystal is formed from a single polar domain.
